# Supplementary material for: Investigating the neuroprotective effect of AAV-mediated β-synuclein overexpression in a transgenic model of synucleinopathy
Source: Sci Rep. 2018 Dec 3;8:17563. doi: 10.1038/s41598-018-35825-2 (PMC6277436; doi:10.1038/s41598-018-35825-2)
Supplement: Supplementary file 1 — Supplementary dataset 1 [file 41598_2018_35825_MOESM1_ESM.docx]

**Supplementary Material**

Title:  **Investigating the neuroprotective effect of AAV-mediated β-synuclein overexpression in a transgenic model of synucleinopathy**

Journal name: Scientific Reports

Authors: Dorian Sargent, Dominique Bétemps, Matthieu Drouyer, Jérémy Verchere, Damien Gaillard, Jean-Noël Arsac, Latifa Lakhdar, Anna Salvetti, Thierry Baron

Corresponding Author: Thierry Baron ([thierry.baron@anses.fr](mailto:thierry.baron@anses.fr))

**Inventory of Supplementary Material:**

Supplementary table 1

Supplementary figure 1

Supplementary figure 2

Supplementary figure 3

Supplementary figure 4

Supplementary figure 5

Supplementary figure 6

Supplementary figure 7

Supplementary figure 8

Supplementary figure 9

Supplementary figure 10

Supplementary figure 11

Supplementary methods

| Age of injection of AAV vectors | Site of injection of AAV vector | AAV vector | Inoculum used for the challenge | Number of mice used for survival analysis |
| --- | --- | --- | --- | --- |
| Day of birth | ICV | AAVβ-syn | M83/M83 | 6 |
| Day of birth | ICV | AAVGFP | M83/M83 | 8 |
| Day of birth | ICV | AAVβ-syn | MSA/M83 | 10 |
| Day of birth | ICV | AAVGFP | MSA/M83 | 7 |
| Day of birth | ICV | AAVβ-syn | - | 11 |
| Day of birth | ICV | AAVGFP | - | 14 |
| 2 months | VTA | AAVβ-syn | M83/M83 | 8 |
| 2 months | VTA | AAVGFP | M83/M83 | 8 |
| 2 months | VTA | AAVβ-syn | MSA/M83 | 8 |
| 2 months | VTA | AAVGFP | MSA/M83 | 9 |
| 2 months | VTA (HD) | AAVβ-syn | M83/M83 | 7 |
| 2 months | VTA (HD) | AAVGFP | M83/M83 | 10 |

**Supplementary table 1.** Summary of animal experiments performed in the study. The total number of mice used for survival analysis is indicated in each of the experimental groups, after AAV inoculations of the two viral vectors by the different strategies described in the study. Brain samples of sick M83 mice were then randomly chosen for analyses by biochemistry or immunohistochemistry. ICV: intracerebroventricular, VTA: ventral tegmental area, HD: High dose of AAV vector injected.


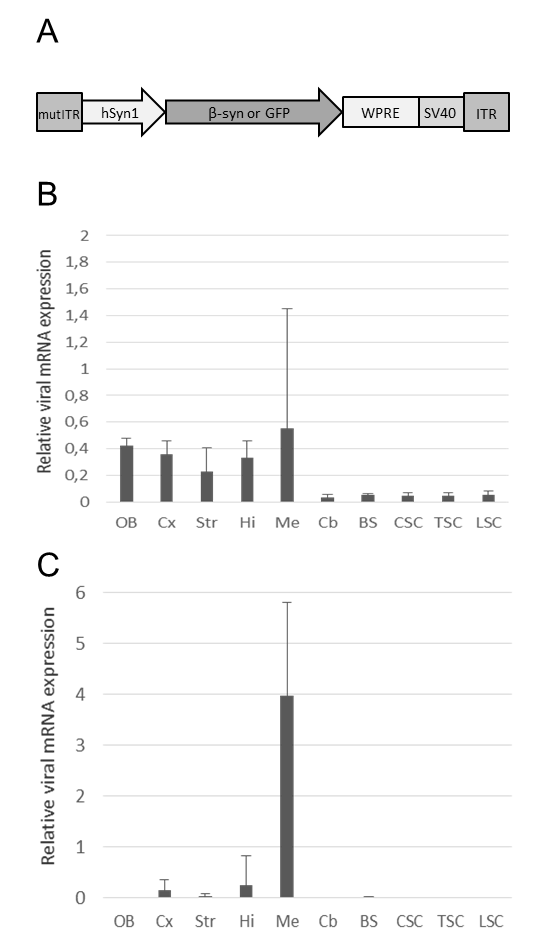


**Supplementary figure 1.** AAVβ-syn expression in wild-type mice after injection by intracerebroventricular route at birth by qRT-PCR. (A) Schematic representation of the genome of the serotype 9 self-complementary AAV (scAAV) vector used in this study. mutITR, mutated Inverted Terminal Repeat; hSyn1, human Synapsin1 promoter; WPRE, Woodchuck Hepatitis Virus Posttranscriptional Regulatory Element; SV40, Simian Virus 40 polyadenylation signal. (B) Wild-type B6C3H neonates were inoculated with 9,38*10^8^ vg of AAVβ-syn per lateral ventricle and euthanized one month later. Levels of human β-syn mRNAs in dissected brains and spinal cord were quantified by qRT-PCR, relative to GAPDH expression (n=6). (C) Two months old wild-type B6C3H mice were inoculated with 3,75*10^8^ vg of AAVβ-syn in the ventral tegmental area (VTA) and euthanized one month later. Levels of human β-syn mRNAs in dissected brains and spinal cords were quantified by qRT-PCR, relative to GAPDH expression (n=6). OB: olfactory bulbs, Cx: cerebral cortex, Str: striatum, Hi: hippocampus, Mes: mesencephalon, BS: brain stem, CSC: cervical spinal cord, TSC: thoracic spinal cord, LSC: lumbar spinal cord. Data are shown as means ± sd.


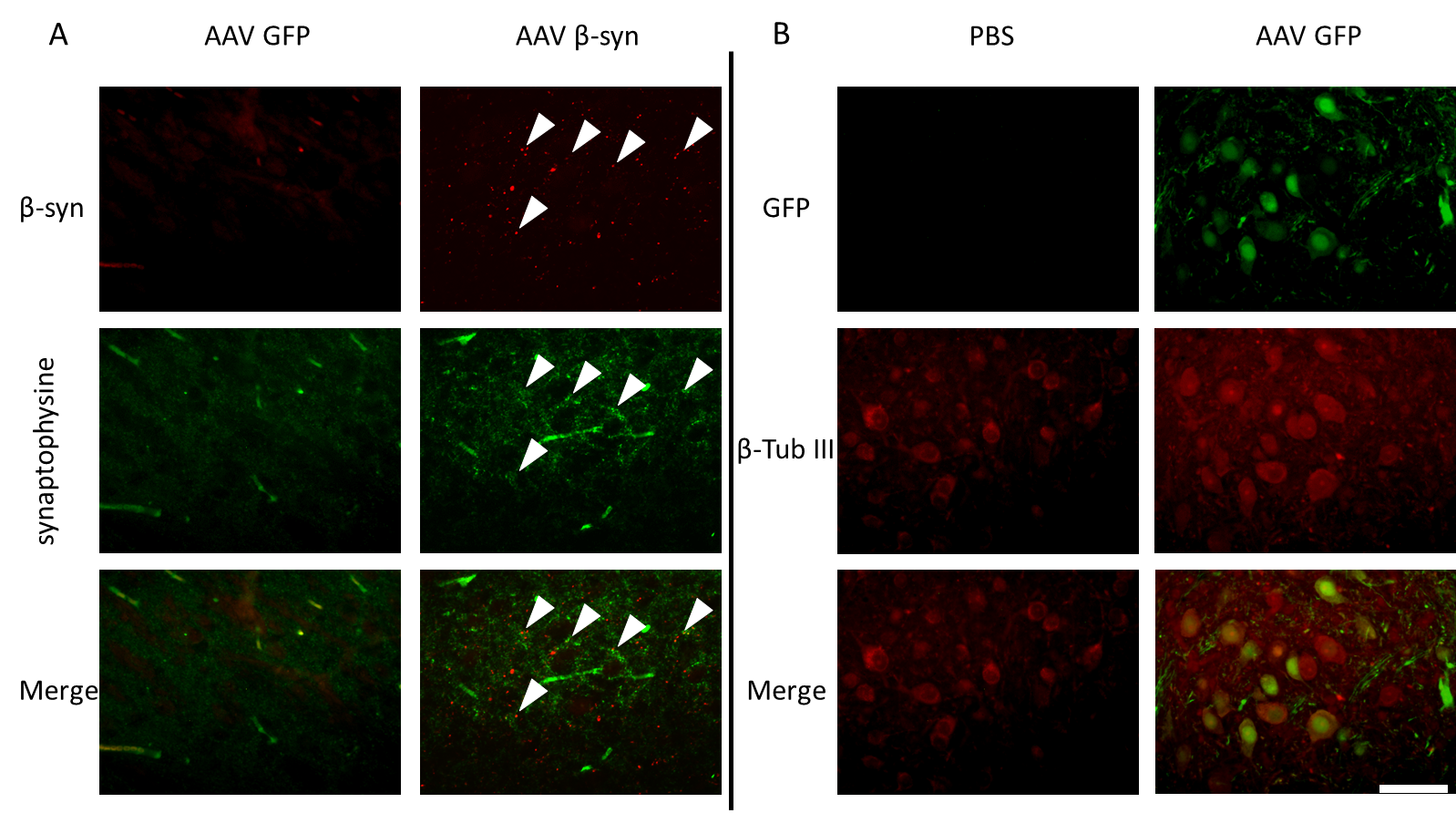


**Supplementary figure 2.** Characterization of the AAVβ-syn or AAVGFP expression after their inoculation in the ventral tegmental area. (A) Co-staining of total β-syn with presynaptic protein synaptophysin in the mesencephalon of sick M83 mice inoculated with AAVβ-syn or AAVGFP in the ventral tegmental area and challenged with MSA/M83 inoculum (mice from the experiment Figure 4E-G). (B) Co-staining of GFP with specific neuronal marker β-tubulin type 3 in the mesencephalon of sick M83 mice inoculated with AAVGFP or PBS and challenged with MSA/M83 inoculum. Scale bar 50µm.


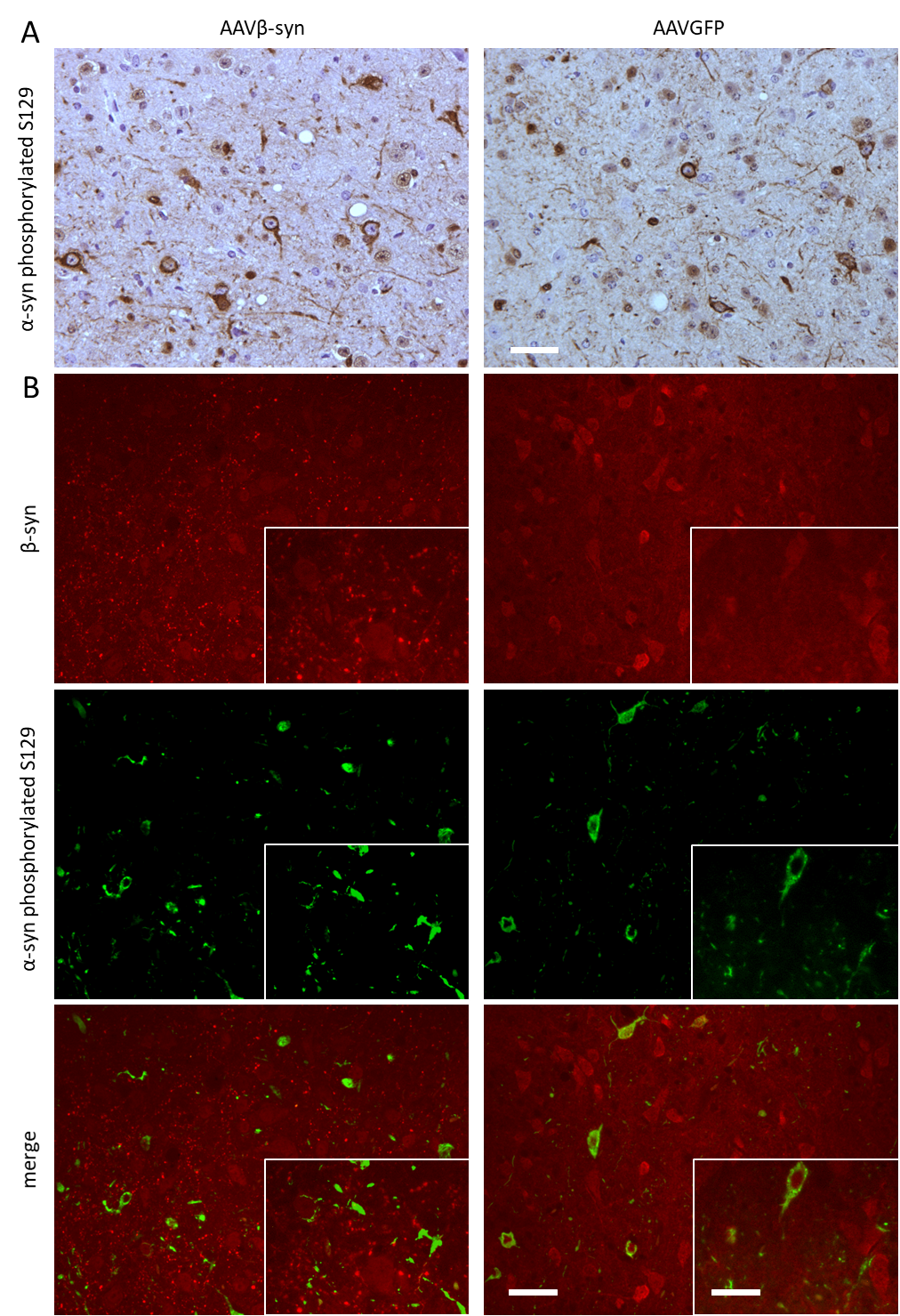


**Supplementary figure 3.** α-syn pathology in sick M83 mice injected with AAVβ-syn or AAVGFP. (A) Detection of α-syn phosphorylated at serine 129 by immunohistochemistry in the mesencephalon of sick M83 mice inoculated with AAVβ-syn or of AAVGFP in the VTA and challenged with MSA/M83 inoculum (mice from the experiment Figure 4E-G). Scale bar 50µm. (B) Co-staining of total β-syn (red) with α-syn phosphorylated at serine 129 (green) by immunofluorescence in the same brain region and same mice as (A). Scale bars 50µm (low magnification) and 25µm (high magnification).


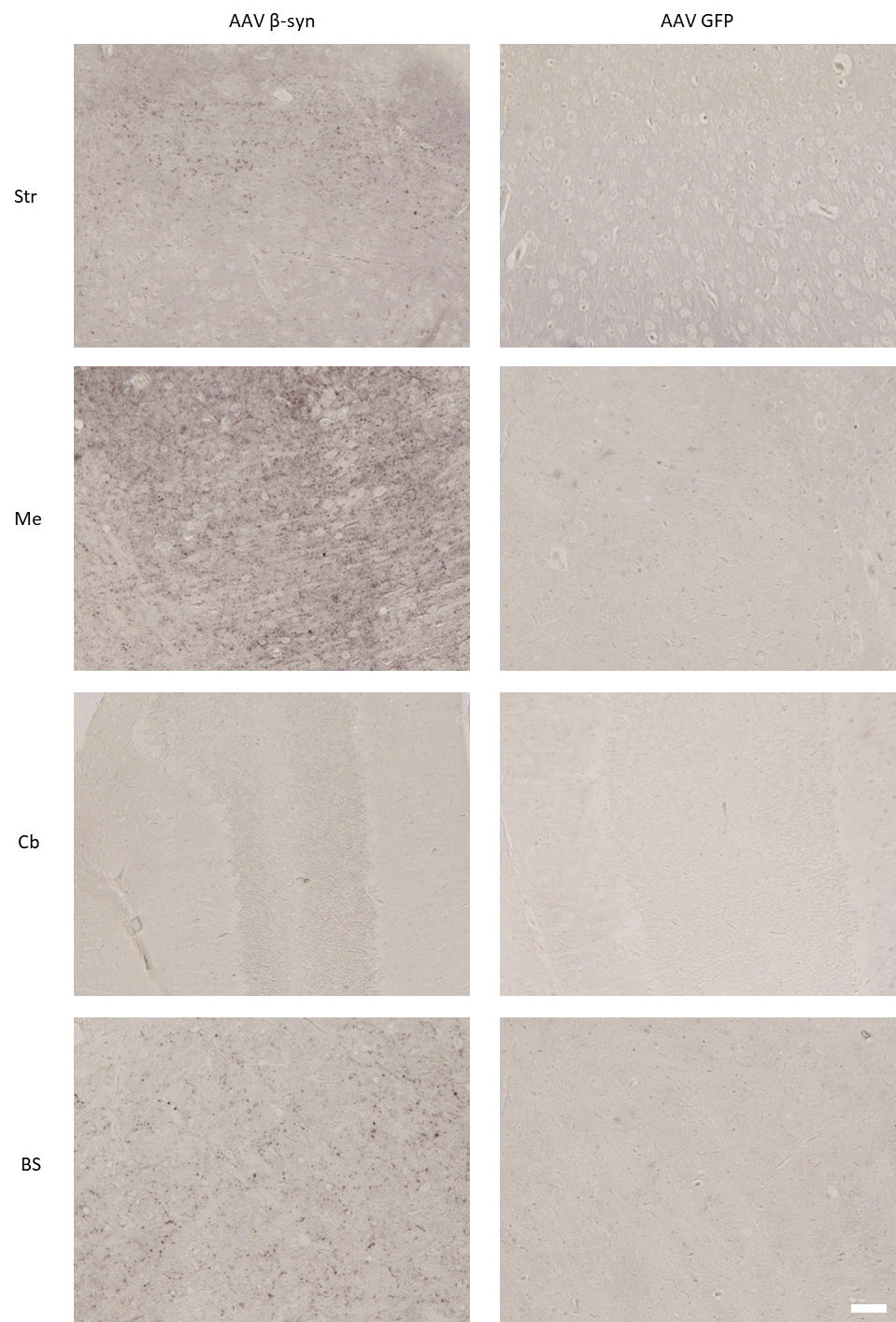


**Supplementary figure 4.** Proteinase K-resistant β-syn staining in brain regions of sick M83 mice inoculated with AAVβ-syn. Total β-syn detection by immunohistochemistry after PK digestion in the striatum (Str), mesencephalon (Me), cerebellum (Cb) and brain stem (BS) of sick M83 mice inoculated with AAVβ-syn or of AAVGFP and challenged with MSA/M83 inoculum (mice from the experiment Figure 4E-G). A Proteinase K-resistant punctate pattern was detected in the Str, Me and BS, but not in the Cb, in which only traces of viral mRNA were detected by qRT-PCR. Scale bar 100µm.


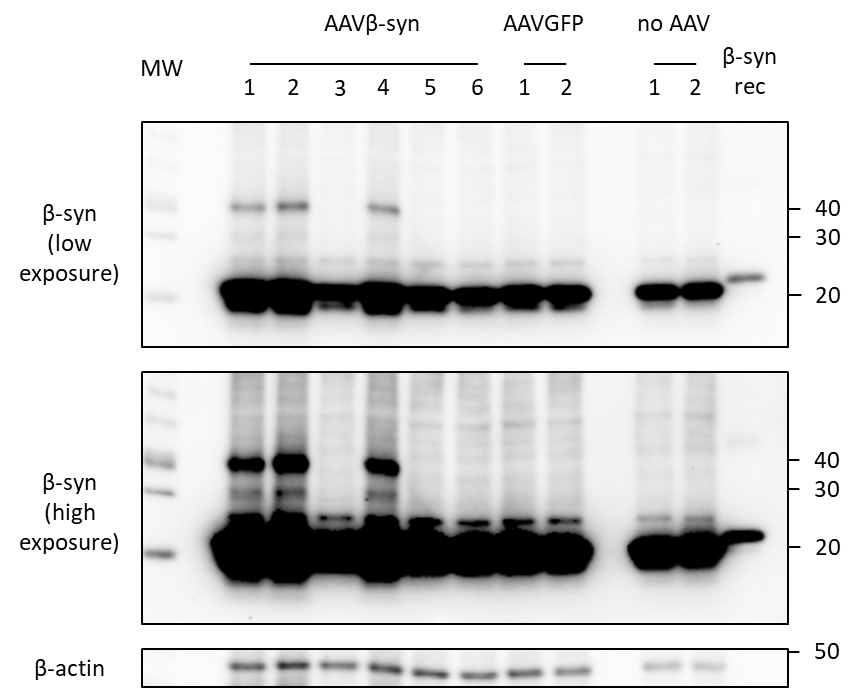


**Supplementary figure 5.** Detection of total β-syn by Western blot in the mesencephalon of sick M83 mice injected with high doses of AAV vectors in the mesencephalon. Homogenates of six mice injected with AAVβ-syn were loaded, comprising 3 mice overexpressing β-syn (mice 1, 2, 4) and 3 mice in which we found no overexpression of β-syn (mice 3, 5, 6), according to ELISA and Western blot results. Two sick M83 mice injected with high doses of AAVGFP and two sick M83 mice not injected with AAV vectors were used as controls. The upper panel shows the detection of β-syn after low exposure (1 minute) and the lower panel shows the same blot with higher exposure (5 minutes). β-actin was detected as a loading control. MW: molecular weight ladder.


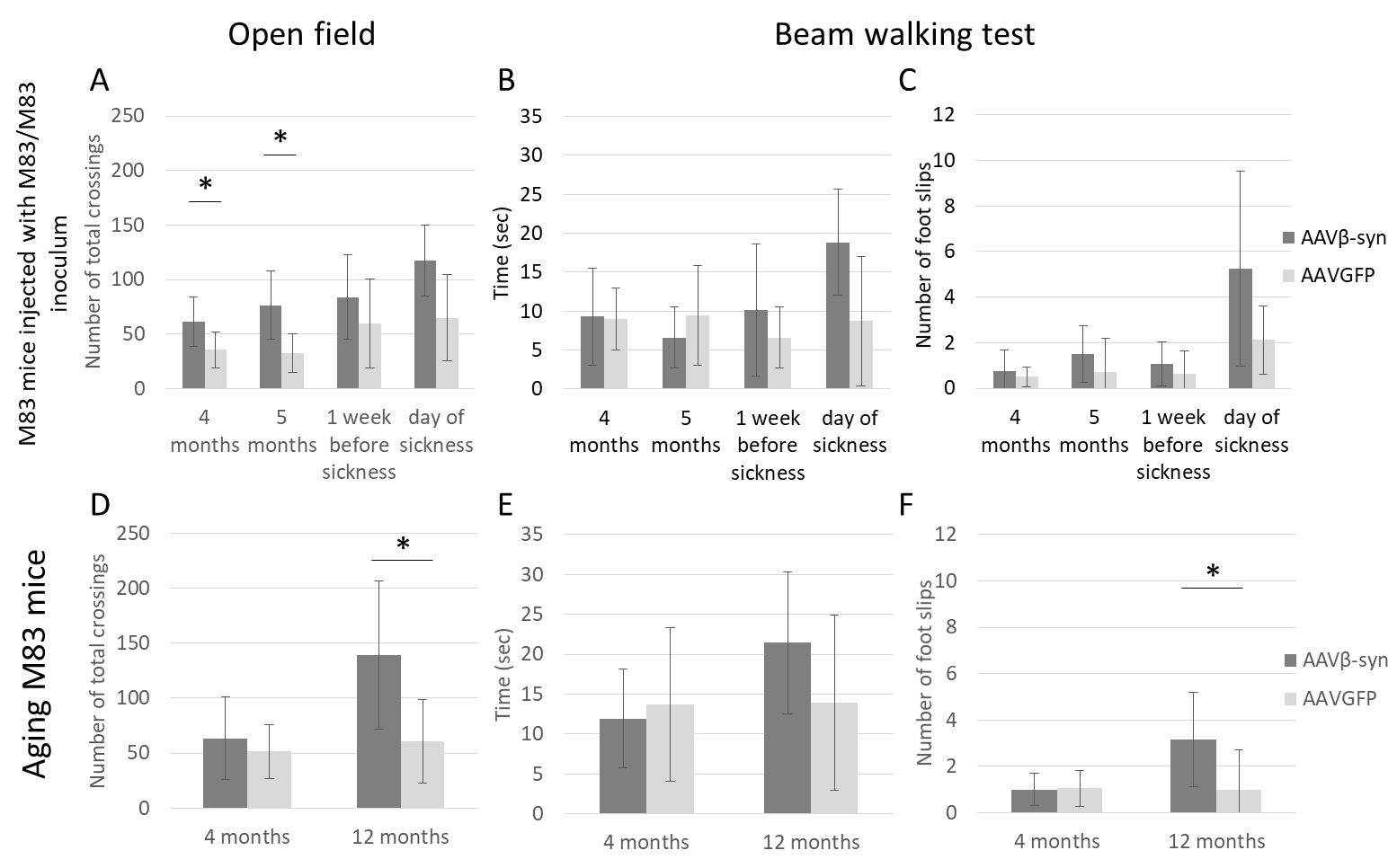


**Supplementary figure 6.** Evaluation of the motricity of M83 mice AAV-inoculated at birth. The global motricity and the motor coordination and balance were assessed on M83 mice injected with AAVβ-syn or AAVGFP in cerebral ventricles at birth and challenged (A-C) or not (D-F) with M83/M83 inoculum (mice from the experiment Figure 1B – D and Figure 3). The age of mice is indicated at the bottom of the histograms. Considering the panels (A-C), mice were tested at 4 months, 5 months and then weekly before we detected symptoms (referred as « day of sickness »). Considering aging mice (without challenge, panels (D-F)), no symptoms was detected at 4 or 12 months. (A, D) Spontaneous activity of M83 mice during an open field test. Total number of crossings during 5 min was counted for each mouse. (B-C, E-F) Beam walking test was performed the same week as the Open field test. The time the mice crossed the beam was measured (B, E) and the number of foot slips was counted (C, F). * p<0,05 according to Student or Wilcoxon test.


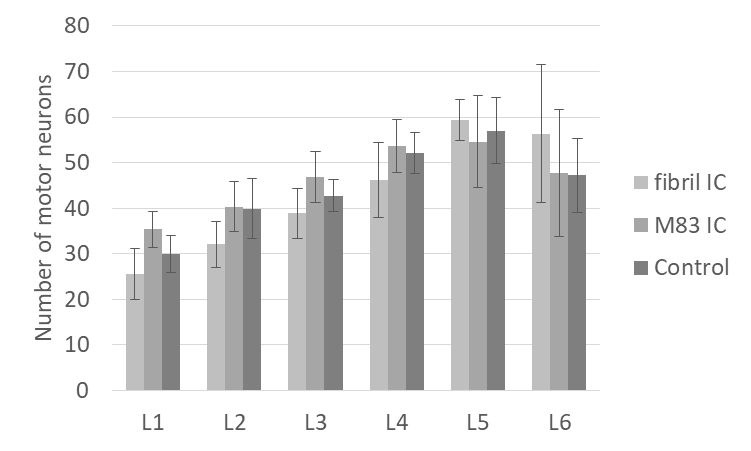


**Supplementary figure 7.** Immunodetection and quantification of choline acetyltransferase-positive motor neurons in the lumbar spinal cord of M83 mice using immunofluorescence. Cell bodies were quantified in the ventral horn of 50µm cross sections of the lumbar spinal cord (L1–L6) of sick M83 mice inoculated with preformed fibrils of recombinant α-syn mutated in A53T (« fibril IC », n=6) or in sick M83 mice injected with brain homogenate from a sick M83 mouse (« M83 IC », n=6) in the striatum. Healthy and non inoculated M83 mice aged three months were used as control (n=3). p>0,05 according to Wilcoxon test.

**
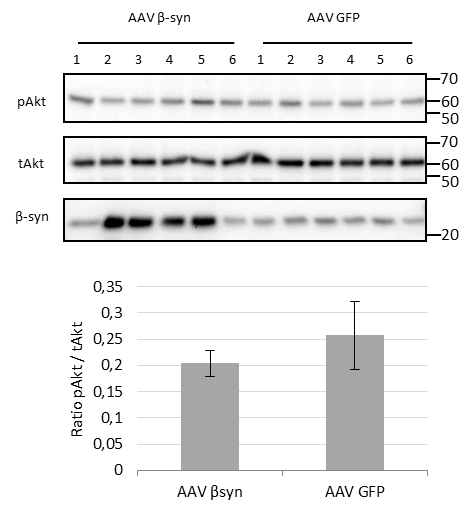
**

**Supplementary figure 8.** β-syn overexpression in the mesencephalon of M83 mice did not activate Akt signaling. Two months old M83 mice were injected with low dose of AAVβ-syn or AAVGFP vectors in the ventral tegmental area (VTA) and challenged one month later by the injection of M83/M83 inoculum in the striatum to accelerate the disease. In this experiment, all mice were euthanized 3 months after the challenge for biochemical analysis (same mice as Figure 5A, B). (A) Detection by Western blot of the Akt protein phosphorylated at the serine 473 (pAkt) and total Akt protein (tAkt) in the mesencephalon. (B) Semi-quantification of the Akt signaling activation, by calculating the ratios pAkt/tAkt. Mice 1 and 6 from the AAVβ-syn group were excluded from the analysis since no β-syn overexpression was detected in these mice. p>0,05 according to Wilcoxon test.


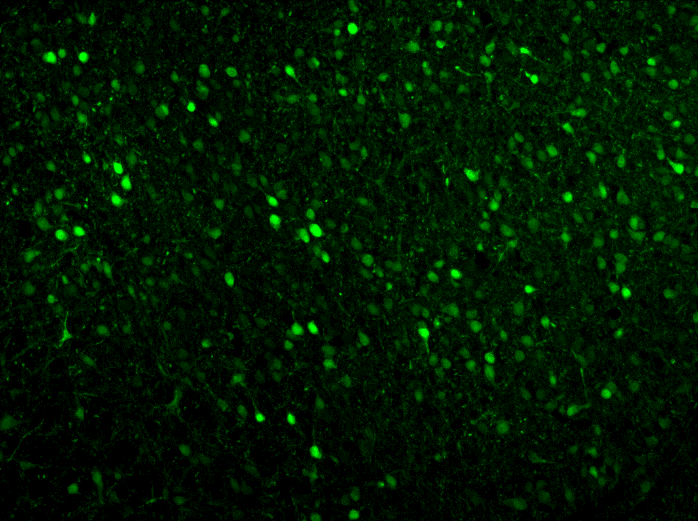

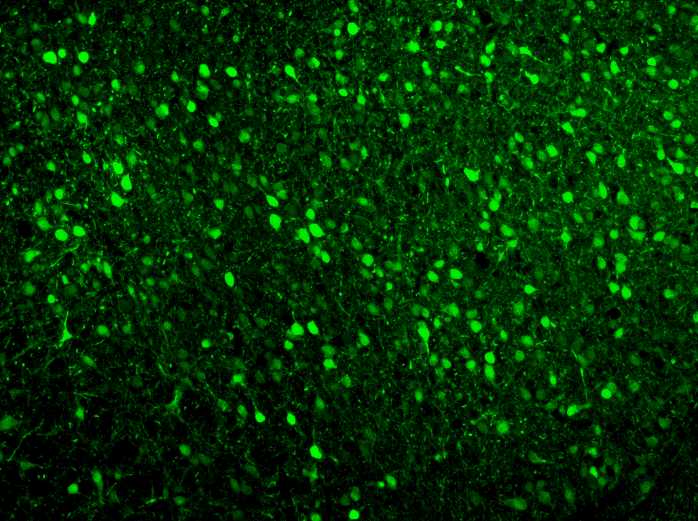


A

B

**Supplementary figure 9.** Immunofluorescence GFP staining in the mesencephalon of a 7 months old sick M83 mouse injected with low dose of AAVGFP in the VTA and challenged one month later by the injection of MSA/M83 inoculum in the striatum (mouse from the experiment Figure 4 E-G). The same picture is shown with 500 ms (A) or 2000 ms (B) exposure time in order to point out the variability of transgene expression in transduced cells.


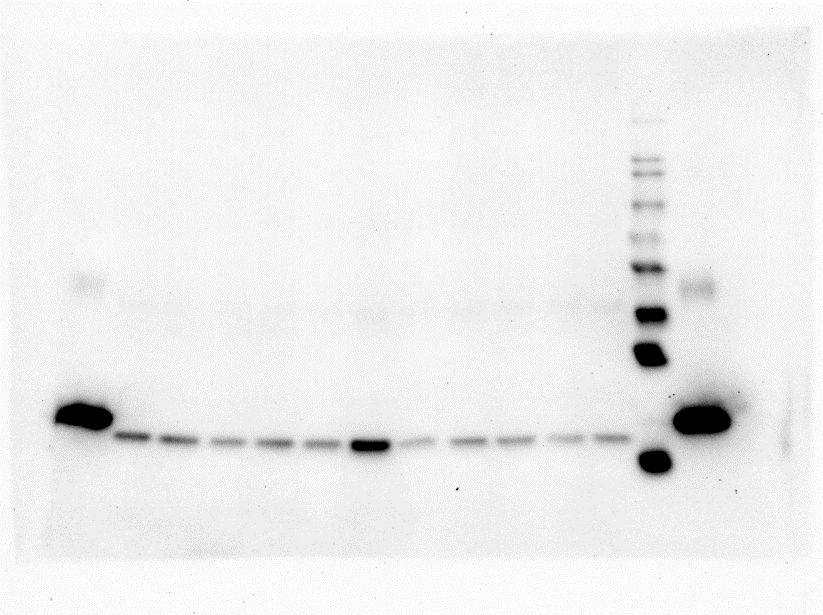

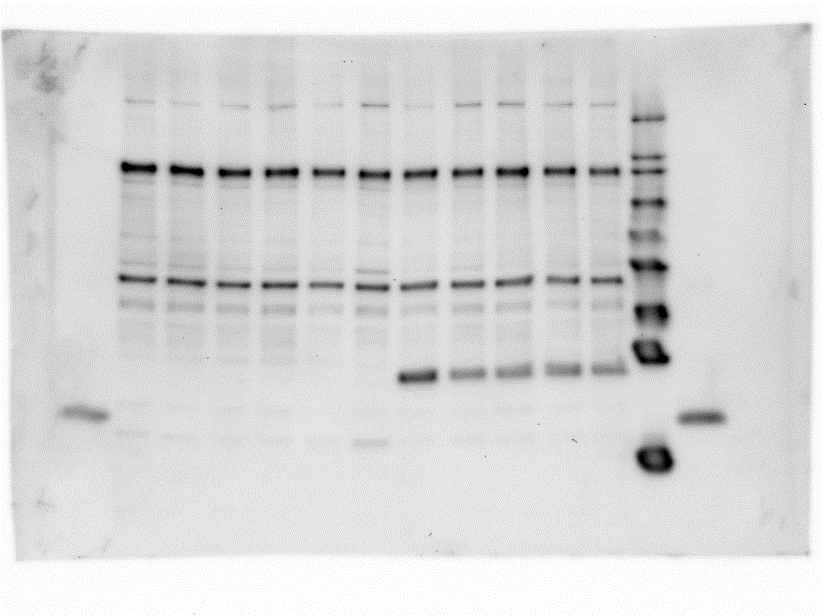

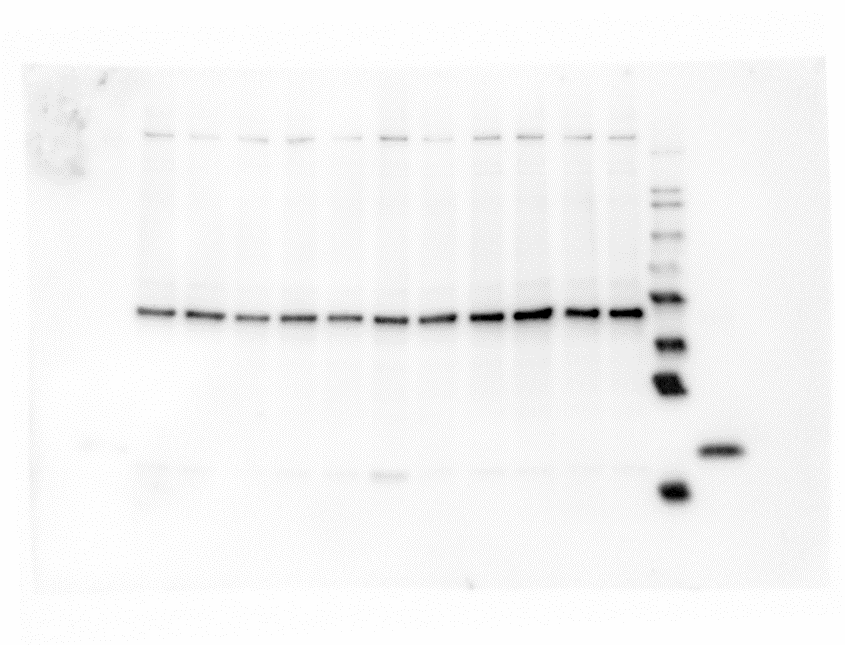


β-syn

β-syn rec

1

2

3

4

5

6

1

2

3

4

5

AAVβ-syn

AAVGFP

β-syn rec

MW

β-actin

GFP

20

30

40

50

60

80

100

120

200

20

30

40

50

60

80

100

120

200

20

30

40

50

60

80

100

120

200

**Supplementary figure 10.** Complete blots showing the detection of β-syn, β-actin and GFP protein using previously characterized antibodies ^1,2^ in the hippocampus of sick M83 mice, 5 to 7 months after ICV injection of AAV (corresponding to cropped blots in Figure 2B). Proteins were detected successively on the same blot. GFP protein was detected at the end of the experiment because the antibody used reveal unspecific bands. MW: molecular weight ladder.


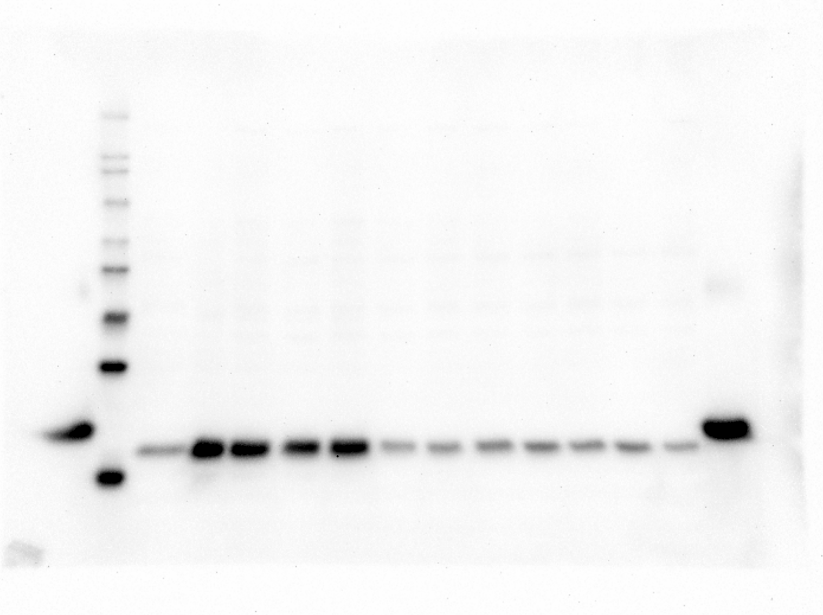

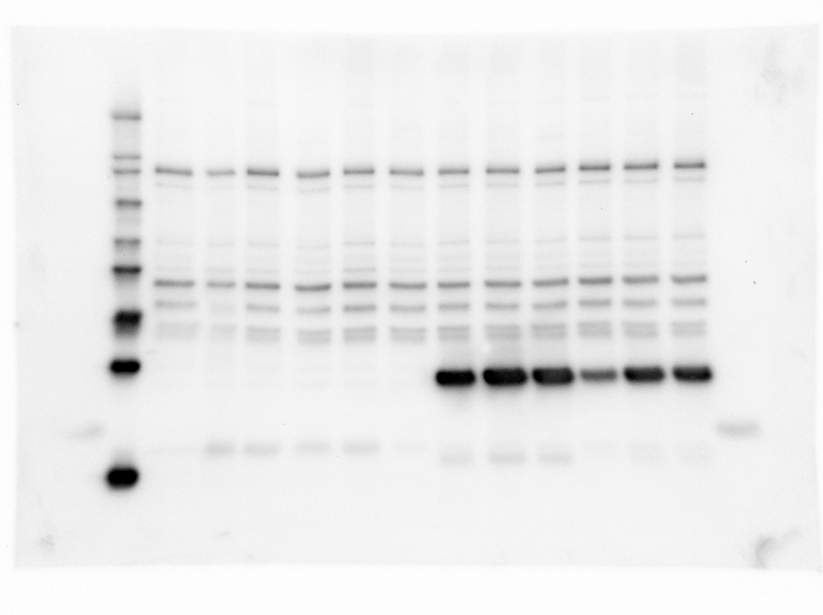

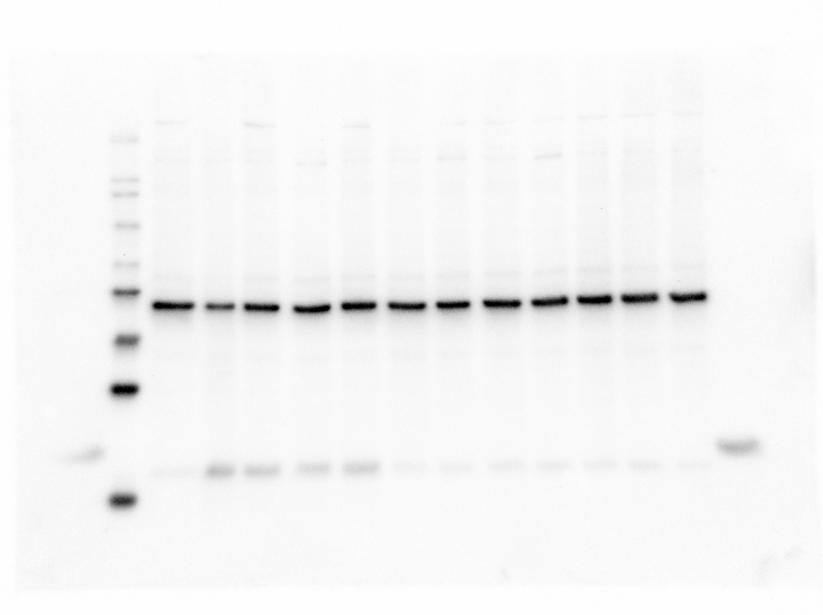


β-syn

β-syn rec

1

2

3

4

5

6

1

2

3

4

5

AAVβ-syn

AAVGFP

β-syn rec

MW

β-actin

GFP

6

20

30

40

50

60

80

100

120

200

20

30

40

50

60

80

100

120

200

20

30

40

50

60

80

100

120

200

**Supplementary figure 11.** Complete blots showing the detection of β-syn, β-actin and GFP protein in the mesencephalon of M83 mice 4 months after inoculation of AAVβ-syn or AAVGFP in the VTA (corresponding to cropped blots in Figure 5B). Proteins were detected successively on the same blot. As before, GFP protein was detected at the end of the experiment because the antibody used reveal unspecific bands. MW: molecular weight ladder.

**Supplementary methods**

*Open field:* M83 mice were tested individually in a Plexiglas box measuring 50 cm×50 cm×20 cm and divided into 25 equal squares of 10 cm. The open field was washed after each session. Mouse was placed in a central square on the open field and was allowed to move freely for 5 min. The total number of square crossings was scored manually by the experimenter.

*Beam walking test:* Mice walked across the beam (80 cm long, 12 mm in diameter) elevated 30 cm above the bench to a goal box. Three trials per day were performed for each mouse, during two days (when the experiment was performed each week, for mice injected with brain extract Supplementary figure 5B-C), or three days (for the 4 months and the 12 months time points, for aging mice, Supplementary figure 5E-F). The time to reach the goal box and the number of foot slips of the hindlimbs were quantified. Mice that fell were returned to the position they fell from, with a maximum time of 60 sec allowed on the beam. We made the statistical analysis with the average of the two best trials obtained the last day of the session per mouse for both parameters.

*Inoculations:* In an additional study (Supplementary figure 5), 2 months old M83 mice were inoculated with 10 µg of preformed fibrils of recombinant α-syn mutated in A53T (kindly provided by Ronald Melki and colleagues ^3^) or with 0.2 mg of brain extract from a sick M83 mouse in the striatum using stereotaxic coordinates (AP: +0,14; ML:+2; DV: -2,75). As for all the experiments, M83 mice were euthanized after the detection of the first symptoms (balance disorders or hind limb paralysis).

*Immunofluorescence:* In order to detect and quantify motor neurons in the lumbar spinal cords (Supplementary figure 5), spinal cords were extracted, and lumbar spinal cords were fixed in 4% paraformaldehyde and embedded in a 4% agarose solution to be cut into serial 50 µm sections using a vibratome. Tissue sections were incubated for 30 min at room temperature in a permeabilization solution 0.5% Tween20 0.5% Triton X-100 in Tris-buffered solution (TBS) (TBSTT) then incubated 45 min in a blocking solution containing 10% BSA in TBSTT. Motor neuron immunodetection was performed using a rabbit choline acetyltransferase (ChAT) primary antibody (ref: ab178850, Abcam, Cambridge, GB) diluted at 1:400 in which tissue sections were incubated overnight at 4°C. After a washing step in 0.5% Triton X-100 in TBS, sections were incubated with an Alexa Fluor anti-rabbit Ig (ref: A21428, Thermo Fisher Scientific, Rockford, USA) for 1 h at room temperature. After washing in TBS, sections were mounted and cell bodies of the ventral horn were manually counted (6 sections per vertebrae of the lumbar spine L1-L6, for each mouse).

**REFERENCES**

1 Lee, Y. *et al.* PINK1 Primes Parkin-Mediated Ubiquitination of PARIS in Dopaminergic Neuronal Survival. *Cell reports* **18**, 918-932, doi:10.1016/j.celrep.2016.12.090 (2017).

2 Betemps, D. *et al.* Alpha-synuclein spreading in M83 mice brain revealed by detection of pathological alpha-synuclein by enhanced ELISA. *Acta Neuropathologica Communications* **2**, 29, doi:10.1186/2051-5960-2-29 (2014).

3 Bousset, L. *et al.* Structural and functional characterization of two alpha-synuclein strains. *Nature communications* **4**, 2575, doi:10.1038/ncomms3575 (2013).
